# A citation analysis of (f)MRI papers that cited Lieberman and Cunningham (2009) to justify their statistical threshold

**DOI:** 10.1371/journal.pone.0309813

**Published:** 2024-09-03

**Authors:** Andy Wai Kan Yeung

**Affiliations:** Oral and Maxillofacial Radiology, Applied Oral Sciences and Community Dental Care, Faculty of Dentistry, The University of Hong Kong, Hong Kong, China; Medical University of Vienna: Medizinische Universitat Wien, AUSTRIA

## Abstract

**Introduction:**

In current neuroimaging studies, the mainstream practice is to report results corrected for multiple comparisons to control for false positives. In 2009, Lieberman and Cunningham published a highly cited report that promotes the use of uncorrected statistical thresholds to balance Types I and II error rates. This paper aims to review recent studies that cited this report, investigating whether the citations were to justify the use of uncorrected statistical thresholds, and if their uncorrected thresholds adhered to the recommended defaults.

**Methods:**

The Web of Science Core Collection online database was queried to identify original articles published during 2019–2022 that cited the report.

**Results:**

It was found that the majority of the citing papers (152/225, 67.6%) used the citation to justify their statistical threshold setting. However, only 19.7% of these 152 papers strictly followed the recommended uncorrected P (P_unc_) < 0.005, k = 10 (15/152, 9.9%) or P_unc_ < 0.005, k = 20 (15/152, 9.9%). Over half (78/152, 51.3%) used various cluster-extent based thresholds with P_unc_, with the predominant choices being P_unc_ < 0.001, k = 50 and P_unc_ < 0.001, k = 10, mostly without justifying their deviation from the default. Few papers matched the voxel size and smoothing kernel size used by the simulations from the report to derive the recommended thresholds.

**Conclusion:**

This survey reveals a disconnect between the use and citation of Lieberman and Cunningham’s report. Future studies should justify their chosen statistical thresholds based on rigorous statistical theory and study-specific parameters, rather than merely citing previous works. Furthermore, this paper encourages the neuroimaging community to publicly share their group-level statistical images and metadata to promote transparency and collaboration.

## Introduction

Low statistical power continues to challenge the neuroscience field, influenced by factors such as sample size and statistical threshold selection [1]. Constraints such as finances and subject availability (e.g., patient group with rare diseases) can limit the sample size, rendering the selection of a statistical threshold, preferably determined before data collection or inspection, an essential aspect of study optimization. This is particularly critical in fMRI analysis, which commonly uses a mass univariate approach, necessitating correction for multiple testing. Uncorrected statistical thresholds can yield a mix of “random activations” and “activation in areas considered to be functionally significant”, while corrected thresholds control for the former but may reduce the latter [2]. Methods such as family-wise error rate (FWE), false-discovery rate (FDR), threshold-free cluster enhancement (TFCE), and non-parametric methods have been introduced for multiple comparison correction [3–5].

Statistical analysis can occur at the voxel level or the cluster level. The former considers a voxel to have significant activation if it passes a pre-determined statistical threshold. The latter involves a two-step process: a voxel-level threshold is selected, and voxels that survive this threshold are then subjected to a cluster-level threshold. Clusters of contiguous voxels that exceed a certain cluster extent (cluster size) determined by this threshold are considered to have significant activation. Consequently, correction for multiple comparisons can occur at the voxel or cluster level. Some experts in the fMRI field have recommended that “if no formal multiple comparisons method is used, the inference must be explicitly labeled ‘uncorrected’” [6], “principled protection against Type I error is an absolute necessity” [7], “inference based on uncorrected statistical results is not acceptable” [8], and “when working with single regions and uncorrected P-values, consider the current discussions on the limitations of P-values” (see the COBIDAS report described by [9]). NeuroImage: Clinical even published an editorial that stated that “Neuroimage: Clinical will not consider submissions that draw inferences from uncorrected P-values” [10].

Lieberman and Cunningham (2009) [11] provided an alternative perspective, demonstrating through simulations that combined intensity and cluster size thresholds, such as uncorrected P < 0.005 with a 10 voxel extent, could balance Types I and II error rates. They suggested that these uncorrected thresholds were equivalent to some form of FDR correction. They also argued that being overly cautious about Type I errors could be detrimental, as it could exclude smaller effects while filtering for the strongest ones. They recommended an emphasis on integrating results from multiple studies through meta-analyses to establish scientific “truths” and self-correct false results over time.

With this background, it becomes intriguing to survey recent literature that, despite being published a decade later, still cites Lieberman and Cunningham (2009). The aim is to examine whether these citations were made to justify the use of uncorrected statistical thresholds and whether their uncorrected thresholds adhered to the recommended defaults. While this may not have been the focus of the original report by Lieberman and Cunningham (2009), this survey provides a helpful reference for researchers to understand the citation practice of the neuroimaging community, and emphasizes the need for future research to justify chosen statistical thresholds based on rigorous statistical theory and study-specific parameters, rather than merely citing previous works.

## Materials and methods

The Web of Science Core Collection online database was queried on 19 September 2023. First, the original paper of Lieberman and Cunningham (2009) [11] was identified by typing its article title into the database. The database showed that it was cited by 1013 papers. Then, the 1013 papers were filtered for those labelled as “article” (original article) and published during 2019–2022. A total of 225 papers were identified. For each of these 225 papers, the following information was extracted manually (see S1 Data):

- The line / sentence citing Lieberman and Cunningham (2009).
- Whether it was cited to justify (f)MRI statistical threshold setting (Yes/No). If No, then data extraction was finished.
- What was the justified statistical threshold: 0 = Used preset FWE-/FDR-/TFCE- corrected threshold; 1 = Uncorrected P (P_unc_) < 0.005, k = 10; 2 = P_unc_ < 0.005, k = 20; 3 = other cluster-extent based thresholds with P_unc_. Thresholds 1 and 2 were general recommendations by Lieberman and Cunningham (2009). If coded 0, then data extraction was finished.
- The exact cluster-extent based threshold with P_unc_ used, if (iii) was coded 3.
- What was the justification for the deviation from the default recommendations, if (iii) was coded 3.
- Whether the voxel size was 3.5 mm × 3.5 mm × 5 mm, the exact voxel size used by Lieberman and Cunningham (2009) during their fMRI simulation (Yes/No).
- The exact voxel size used, if (vi) was coded No.
- Whether the smoothing kernel (FWHM) was 6 mm, as used by Lieberman and Cunningham (2009) during their fMRI simulation (Yes/No).
- The exact FWHM used, if (viii) was coded No.

## Results

The coded data sheet was provided as the S1 Data. It was revealed that two-thirds of the citing papers (152/225, 67.6%) cited Lieberman and Cunningham (2009) to justify their (f)MRI statistical threshold setting. Among these 152 papers, 19.7% followed the exact recommendations of either P_unc_ < 0.005, k = 10 (15/152, 9.9%) or P_unc_ < 0.005, k = 20 (15/152, 9.9%); whereas 28.9% (44/152) used preset FWE- / FDR- / TFCE- corrected threshold. The remaining articles (78/152, 51.3%) used a variety of cluster-extent based thresholds with P_unc_, with the predominant choices being P_unc_ < 0.001, k = 50 and P_unc_ < 0.001, k = 10 (Table 1). Please refer to S1 Table for the frequency count of all variants.

**Table 1:** The most common cluster-extent based thresholds with P_unc_, deviated from default recommendations, used in 78 papers.

Statistical threshold	Number of papers
P_unc_ < 0.001, k = 50	11
P_unc_ < 0.001, k = 10	11
P_unc_ < 0.001, k = 20	8
P_unc_ < 0.005, k = non-stationary	4
P_unc_ < 0.001, k = 100	4
P_unc_ < 0.001, k = 30	4
P_unc_ < 0.005, k = 100	3
P_unc_ < 0.005, k = 0	3
P_unc_ < 0.001, k = non-stationary	3
P_unc_ < 0.001, k = 19	3
P_unc_ < 0.001, k = 0	3

k, cluster extent. P_unc_, uncorrected P.

The common justifications for the deviation from the default threshold recommendations were being more stringent (7/78, 9.0%) and being based on prior studies (5/78, 6.4%). All threshold variants with non-stationary cluster extent, together with some other variants with a fixed cluster extent, had their cluster extent determined by fMRI software (14/78, 17.9%), such as 3dClustSim, AlphaSim, BrainVoyager, SPM_ClusterSizeThreshold, VBM-8, and xjview, mostly with the Monte Carlo simulation approach. Among these 14 papers, 5 did not use P_unc_ < 0.005: three of them did not provide a justification, one was based on prior studies, and one mentioned that it was more stringent. In other words, 9 papers used P_unc_ < 0.005 with cluster size properly determined by fMRI software (Monte Carlo simulation). The majority of the papers that used a cluster-extent based threshold deviation from the default did not provide any justifications (51/78, 65.4%). As reported in Table 1, one paper (1/78, 1.3%) did not report the exact cluster-extent based threshold, and hence justification was not determined.

**Fig 1:**
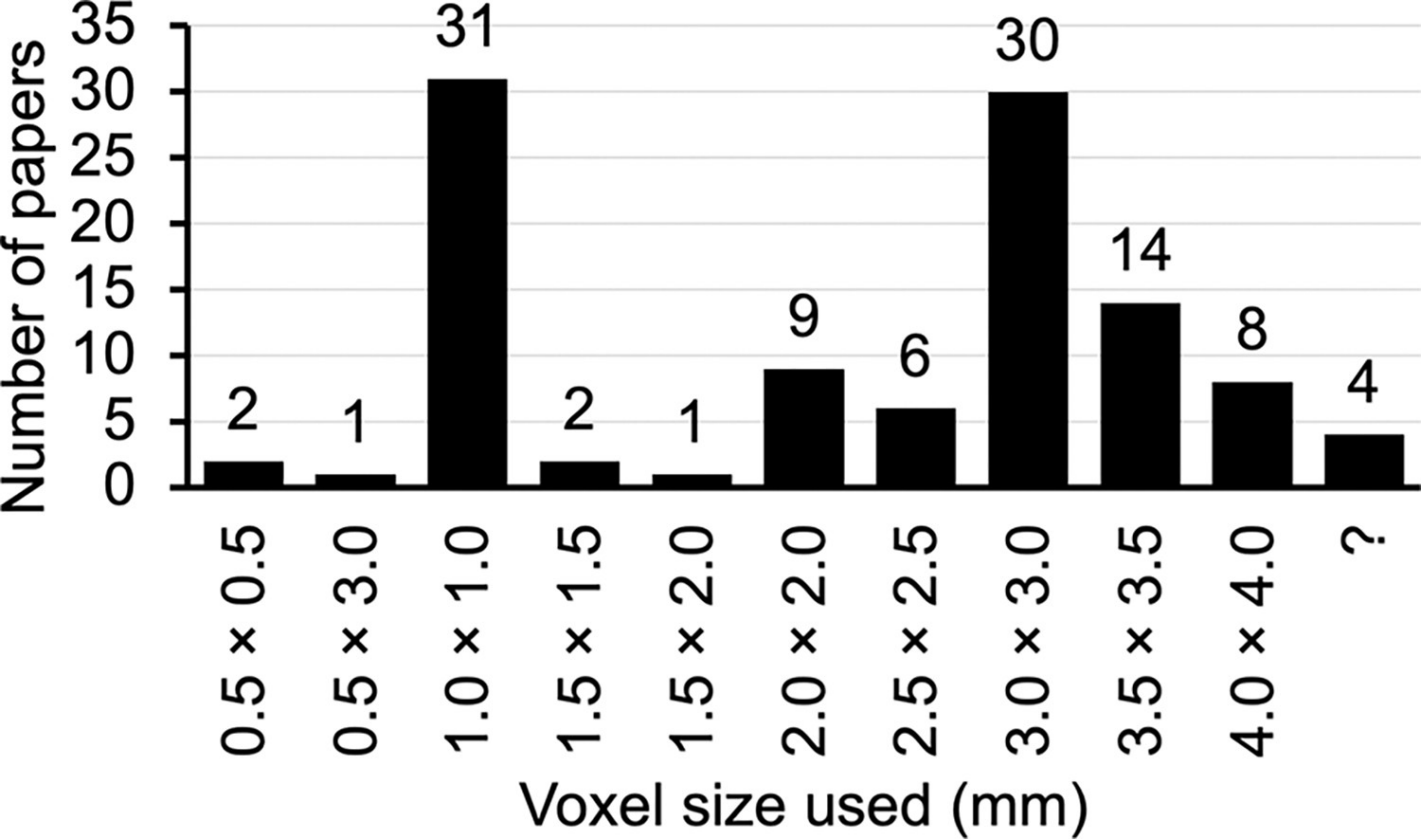
Frequency breakdown of voxel size (rounded up to the nearest 0.5) used in 108 papers. Slice thickness was not considered in this chart.

Among the 108 papers that used cluster-extent based thresholds (regardless of following the default recommendations or not), only 1 had the same voxel size of 3.5 mm × 3.5 mm × 5 mm used by Lieberman and Cunningham (2009). When the voxel size was rounded up to the nearest 0.5, then 1 more paper matched with this voxel size. Fig 1 lists a frequency breakdown of voxel size used by these 108 papers. Though 14 papers had a voxel size of 3.5 mm × 3.5 mm, most of them were isotropic or nearly isotropic with 3.0–4.0 mm slice thickness instead of 5 mm. In other words, only 2 papers (2/108, 1.9%) had the voxel size meeting the assumption from Lieberman and Cunningham (2009).

**Fig 2:**
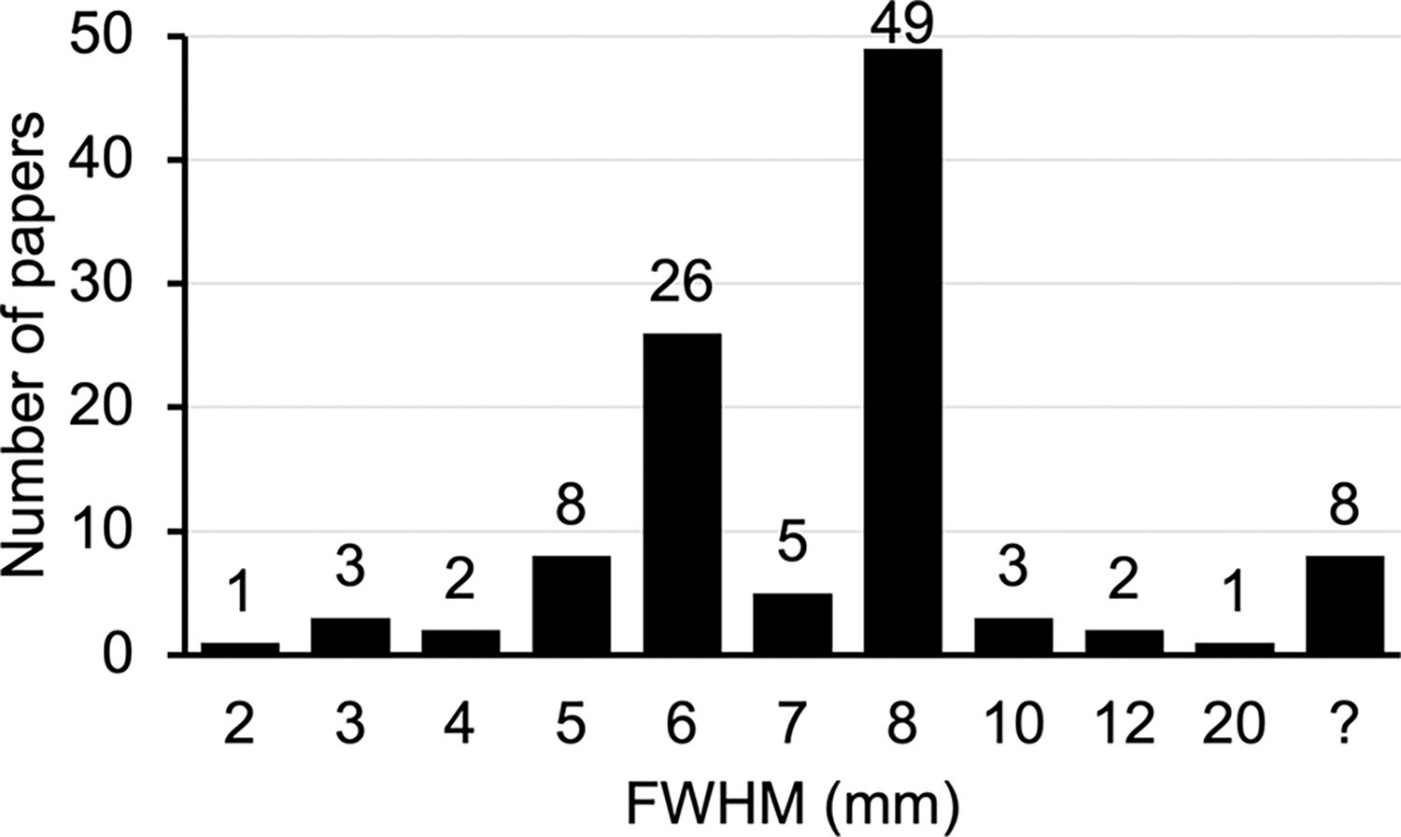
Frequency breakdown of smoothing kernel used in 108 papers.

In terms of smoothing kernel size, 26 papers (26/108, 24.1%) used a FWHM of 6 mm, the same size used by Lieberman and Cunningham (2009). Nearly 70% papers (74/108, 68.5%) used a different FWHM (Fig 2), and 7.4% (8/108) did not report the FWHM. Smoothing was most frequently done with 8 mm FWHM.

In summary, none of the 108 papers that used cluster-extent based threshold simultaneously matched with the recommended statistical threshold(s), exact voxel size and smoothing kernel size used by Lieberman and Cunningham (2009).

## Discussion

This study revealed that approximately two-thirds of the surveyed papers (152/225) cited Lieberman and Cunningham (2009) to justify their statistical threshold used in (f)MRI data analysis. Among them, only one-fifth (30/152) used the default recommended thresholds, but none of them used both the voxel size and smoothing kernel size assumed by Lieberman and Cunningham (2009) in their simulation with AlphaSim. Over half (78/152) used a variety of cluster-extent based thresholds with P_unc_, among which the majority did not provide any justifications regarding the use of the threshold. The remaining three-tenth (28.9%) of the papers (44/152) used preset FWE- / FDR- / TFCE- corrected threshold.

It is understood that each study would come with its own voxel size, number of slices, widths of smoothing kernels, and so on, all of which depend on the needs of each particular study. Therefore, it is very important for researchers to explain or elaborate on how they have determined their statistical thresholds, especially if they deviated from established, recommended, or exampled thresholds. It should be reiterated that the purpose of this study is not to justify or refute the use of a particular statistical inference procedure but to analyze the reasons behind the citations of Lieberman and Cunningham (2009) and to provide general recommendations for better practices in the field.

There were two schools of thoughts in the neuroimaging community. On one end, some researchers strongly advocated for the use of corrected statistical thresholds such as FWE and FDR corrections to minimize false positive results (Type I error) [7, 8]. On the other end, some researchers advocated for more liberal thresholds including uncorrected thresholds to produce a desirable balance between Types I and II error rates [11]. In particular, Lieberman and Cunningham (2009) argued that false positive results could be disregarded when subsequent replication studies and meta-analyses were conducted to report new or more robust results, but false negative results could not be aggregated and hence not self-correctable. This notion of a good “balance” between Type I (false positive) and II (false negative) errors / sensitivity (true positive rate) and specificity (true negative rate) was well-received by the audience, as 67 of the 225 analyzed papers (29.8%) explicitly mentioned this “balance” in their citing lines. However, these citing papers, particularly those using customized uncorrected thresholds, usually did not explain how they came up with their own choice of threshold based on their methodological parameters such as voxel size, number of slices, and so on. Regardless, it is advisable to report corrected results based on methods that guarantee control of the desired error rate under a given set of assumptions (Table 2), instead of results based on arbitrary thresholds. Meanwhile, researchers should be encouraged to share group-level statistical maps (including unthresholded images) and metadata on publicly accessible repositories such as NeuroSynth [12], NeuroVault [13], and OpenNeuro [14].

**Table 2:** Common statistical correction procedures and their assumptions. Contents were based on [15–19].

Correction procedure	Assumptions
Permutation	Exchangeability: the labels or assignments of the data points can be freely permuted without affecting the null hypothesis
Bootstrap	Data points are independently and identically distributed
Gaussian random field (GRF)	Data follow a multivariate Gaussian distribution, have an isotropic spatial autocorrelation structure, and adopt a stringent cluster forming threshold for the approximations

In practice, false positive results might affect subsequent studies, especially if region-of-interest (ROI) analysis was performed instead of whole-brain analysis. In these studies, the ROIs would be selected usually based on the significant results from previous studies, with vague statements on how the past results were relevant to the present investigation settings [20]. Besides, a recent survey on activation likelihood estimation (ALE) meta-analyses found that only 30.3% of them met the recommendation of having at least 17 experiments per analysis [21], rendering the meta-analytic results potentially vulnerable to false positive results from individual studies. Meanwhile, although the original simulation with AlphaSim by Lieberman and Cunningham (2009) reported an equivalence to P_FDR_ < 0.05, a subsequent simulation study reached a different conclusion [22]. In that study, an even more stringent threshold of P_unc_ < 0.001 and k = 10 would equate to P_FWE_ = 0.6–0.9 with common fMRI software except the FLAME 1 module of FSL. Moreover, 3dClustSim (successor of AlphaSim) was found to use a very low group smoothness compared to other software and contain a bug that underestimated the severity of the multiplicity correction [22]. As different datasets have different smoothness properties and site effects, the applicability of the simulation by Lieberman and Cunningham (2009) to other datasets could be questionable. Because of that, some neuroscience journals (e.g., NeuroImage: Clinical) rejected manuscripts that solely reported results based on uncorrected statistics [10]. Regardless of the reasons, studies reporting only uncorrected results seemed to be on a decline and they formed the minority of the literature, with an estimation of as few as 4.4% of task-based fMRI papers published in 2017 [3].

Another issue found from the analyzed papers was that some non-(f)MRI papers cited Lieberman and Cunningham (2009) to justify their choice of statistical thresholds, such as for SDM meta-analysis, ALE meta-analysis, and original studies using EEG, FDG-PET, fNIRS, or MEG (see S1 Data). The suitability of the recommended thresholds was doubtful as they were devised from fMRI data simulations. For neuroimaging meta-analysis, researchers may refer to a “ten simple rules” guideline for statistical recommendations [23], as a recent literature survey pointed out that statistical threshold was the third most common reason for citing the guideline [24]. For EEG and MEG studies, researchers may refer to another recent guideline paper that mentioned: “results must be corrected for multiple testing and comparisons (for example, full-brain analyses or multiple feature and component maxima)” [25]. For fNIRS, it was suggested that: “when a single channel or region of interest is analyzed based on a priori knowledge, statistical inference can be made based on an uncorrected p-value. However, if statistical analysis is performed on multiple channels, regions, or network components, a statistical inference should be adjusted to reduce the risk of the Type I error (false positive) by correcting for multiple comparisons” [26]. The determination of optimal statistical thresholds by these non-(f)MRI studies was beyond the scope of this work, but the above-mentioned examples highlighted that they should cite the more relevant references but not Lieberman and Cunningham (2009).

## Conclusions

Based on this work, it was found that Lieberman and Cunningham (2009) was still frequently cited by many researchers one decade after its publication to justify their choice of statistical thresholds. However, only a small percentage of such citing papers actually used the recommended uncorrected thresholds. A variety of cluster-extent based thresholds with P_unc_ were used, mostly without explicit explanations on why they deviated from the default settings or how they selected the altered settings based on their own experimental parameters. This work highlights the improper citation practice and the need for more rigorous and transparent statistical practice in the neuroimaging community. Future studies should use correction methods that have been demonstrated to target a particular error rate based on sound statistical theories, such as permutation, bootstrap, and Gaussian random field (GRF), under a set of evaluable assumptions. Furthermore, researchers should consider publicly posting their group-level statistical images and metadata on platforms like OpenNeuro, NeuroSynth, and NeuroVault to promote open data sharing and collaboration in the neuroimaging community.

## Supporting information

S1 Data Data sheet for the analysis. (XLSX)

S1 Table The full variety of cluster-extent based thresholds with P_unc_, deviated from default recommendations, used in 78 papers. (DOCX)
