# Supplementary material for: A citation analysis of (f)MRI papers that cited Lieberman and Cunningham (2009) to justify their statistical threshold
Source: PLoS One. 2024 Sep 3;19(9):e0309813. doi: 10.1371/journal.pone.0309813 (PMC11371206; doi:10.1371/journal.pone.0309813)
Supplement: S1 Table — (DOCX) [file pone.0309813.s002.docx]

**S1 Table. The full variety of cluster-extent based thresholds with P_unc_, deviated from default recommendations, used in 78 papers.**

| Statistical threshold | Number of papers |
| --- | --- |
| P_unc_ < 0.005, k = non-stationary | 4 |
| P_unc_ < 0.005, k = 224 | 1 |
| P_unc_ < 0.005, k = 150 | 1 |
| P_unc_ < 0.005, k = 100 | 3 |
| P_unc_ < 0.005, k = 54 | 1 |
| P_unc_ < 0.005, k = 50 | 2 |
| P_unc_ < 0.005, k = 46 | 1 |
| P_unc_ < 0.005, k = 30 | 1 |
| P_unc_ < 0.005, k = 25 | 1 |
| P_unc_ < 0.005, k = 18 | 1 |
| P_unc_ < 0.005, k = 13 | 1 |
| P_unc_ < 0.005, k = 12 | 1 |
| P_unc_ < 0.005, k = 5 | 2 |
| P_unc_ < 0.005, k = 0 | 3 |
| P_unc_ < 0.0025, k = 250 | 1 |
| P_unc_ < 0.001, k = non-stationary | 3 |
| P_unc_ < 0.001, k = 200 | 2 |
| P_unc_ < 0.001, k = 100 | 4 |
| P_unc_ < 0.001, k = 50 | 11 |
| P_unc_ < 0.001, k = 30 | 4 |
| P_unc_ < 0.001, k = 25 | 1 |
| P_unc_ < 0.001, k = 20 | 8 |
| P_unc_ < 0.001, k = 19 | 3 |
| P_unc_ < 0.001, k = 10 | 11 |
| P_unc_ < 0.001, k = 8 | 1 |
| P_unc_ < 0.001, k = 4 | 1 |
| P_unc_ < 0.001, k = 0 | 3 |
| P_unc_ < 0.0005, k = 19 | 1 |
| Unclear | 1 |

k, cluster extent. P_unc_, uncorrected P.
